# Comparison of the Genetic Organization, Expression Strategies and Oncogenic Potential of HTLV-1 and HTLV-2

**DOI:** 10.1155/2012/876153

**Published:** 2011-12-29

**Authors:** Francesca Rende, Ilaria Cavallari, Maria Grazia Romanelli, Erica Diani, Umberto Bertazzoni, Vincenzo Ciminale

**Affiliations:** ^1^Department of Oncology and Surgical Sciences, The University of Padova, 35128 Padova, Italy; ^2^Department of Life and Reproduction Sciences, University of Verona, 37134 Verona, Italy

## Abstract

Human T cell leukemia virus types 1 and 2 (HTLV-1 and HTLV-2) are genetically related complex retroviruses that are capable of immortalizing human T-cells *in vitro* and establish life-long persistent infections *in vivo*. In spite of these apparent similarities, HTLV-1 and HTLV-2 exhibit a significantly different pathogenic potential. HTLV-1 is recognized as the causative agent of adult T-cell leukemia/lymphoma (ATLL) and tropical spastic paraparesis/HTLV-1-associated myelopathy (TSP/HAM). In contrast, HTLV-2 has not been causally linked to human malignancy, although it may increase the risk of developing inflammatory neuropathies and infectious diseases. The present paper is focused on the studies aimed at defining the viral genetic determinants of the pathobiology of HTLV-1 and HTLV-2 through a comparison of the expression strategies and functional properties of the different gene products of the two viruses.

## 1. Introduction

Human T-cell leukemia virus types 1 and 2 (HTLV-1 and HTLV-2) are related deltaretroviruses [1] with similar genetic organization [2–7]. The two viruses share an average 65% homology at the nucleotide level, with higher conservation in the gag, pol, env, and tax/rex genes and lower in the long terminal repeats (LTR), protease, and proximal “X region,” a region located at the 3′ end of the genome.

 Although both viruses immortalize T cells in culture and establish life-long persistent infections *in vivo*, they exhibit a significantly different pathogenic potential. HTLV-1 is recognized as the causative agent of adult T-cell leukemia/lymphoma (ATLL) and tropical spastic paraparesis/HTLV-1-associated myelopathy (TSP/HAM). In contrast, HTLV-2 has not been causally linked to human malignancy. However, large cohort studies revealed that HTLV-2 infection may be associated with lymphocytosis, increased risk of developing inflammatory neuropathies, infectious diseases, and with increased all-cause mortality [4–6, 8]. Furthermore, coinfection with HTLV-2 plays an important role in the progression of HIV-infected patients to AIDS [9].

 In the present paper, we will focus on the discussion of studies aimed at comparing the expression strategies, regulation, and pathogenic properties of HTLV-1 and HTLV-2.

## 2. The Genetic Organization and Expression Strategy of HTLV-1 and HTLV-2

**Figure 1:**
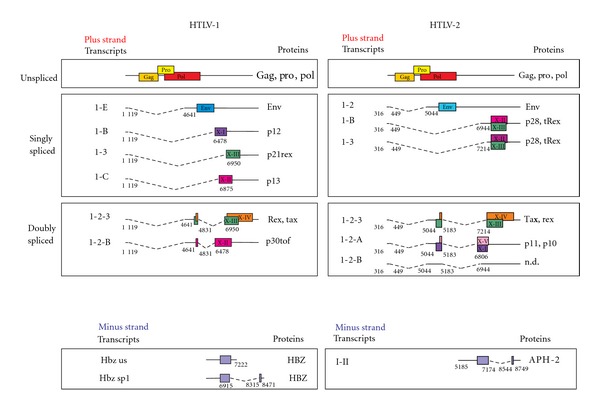
Comparison of the organization, alternative splicing, and coding potential of HTLV-1 and HTLV-2 mRNAs. Exon composition and coding potential of HTLV-1 and HTLV-2 alternatively spliced mRNAs. ORFs are indicated by colored boxes. Splice sites are indicated by numbers. n.d.: not determined.

Like other complex deltaretroviruses, HTLV-1 and HTLV-2 are characterized by the presence of the “X region,” in addition to the LTR and the gag, pol, and env genes present in all retroviruses. The coding potential of the HTLV genomes is greatly enhanced by several expression strategies that include ribosomal frame shifting (which generates a Gag-Pro-Pol polyprotein) and alternative splicing (which produce distinct mRNAs coding for Env and the proteins coded by the X region) (Figure 1); in addition, some of the alternatively spliced transcripts are polycistronic [3, 10, 11]. Recent studies showed that HTLV-1 and HTLV-2 also produce complementary-strand mRNAs, transcribed by promoters in the 3′ LTR; these genes were termed HBZ (HTLV-1 bZIP factor) and APH2 (anti-sense protein of HTLV-2), respectively [12, 13].

 Transcription from the 5′LTR promoter generates 3 major size classes of mRNAs (Figure 1): (a) full-length (9 kb) genomic mRNA, coding for Gag-Pro-Pol; (b) 4 kb singly-spliced mRNAs, coding for the envelope glycoproteins (Env); (c) mRNAs of approximately 2 kb encoding proteins of the X region. The X regions of HTLV-1 and HTLV-2 encode, respectively, four and five major open reading frames (ORFs), termed x-I through x-V. The x-III and x-IV ORFs code for the essential regulatory proteins Tax and Rex that are produced from a dicistronic doubly spliced mRNA containing exons 1, 2, and 3. Tax and Rex provide, respectively, a positive feedback loop that drives transcription of the viral genome [2] and a posttranscriptional regulatory loop enhancing the nuclear export and expression of a subset of mRNAs [14, 15] (see paragraphs below for details). Other transcripts of the 2 kb class encode the accessory proteins of the X region; these transcripts include singly spliced mRNAs coding for p21rex, p12, and p13 (HTLV-1), tRex and p28 (HTLV-2) and doubly-spliced mRNAs, coding for the accessory proteins p30tof (HTLV-1), p10, and p11 (HTLV-2) [3, 10, 11]. Most of the regulatory and accessory proteins of the two viruses were shown to share structural and functional homologies, while p13 and p8 appear to be unique for HTLV-1 and p11 for HTLV-2 (Table 1).

 The discovery of the complex coding potential of deltaretroviruses also raised the question as to whether the different genes are expressed with a particular temporal sequence and whether different patterns of viral gene expression are associated with different disease outcomes. The temporal sequence of HTLV-1 gene expression has recently been investigated using splice site-specific real-time RT-PCR (qRT-PCR) in an *ex vivo* virus reactivation model based on the depletion of CD8+ T cells from unstimulated peripheral blood mononuclear cells (PBMCs) isolated from HTLV-1-infected patients [16]. The results indicated a “two-phase” kinetics with tax/rex expression preceding that of other viral transcripts, a finding that is consistent with the key role of Tax as a master regulator driving overall viral gene expression and suggests an “early-late” switch in HTLV-1 gene expression. Studies in HeLa cells transfected with HTLV-1 molecular clones demonstrated the strict Rex-dependency of this “two-phase” kinetics [16]. Mathematical modelling revealed that the observed “two-phase” kinetics was critically dependent on a delay of Rex function compared to Tax [17], a prediction that was supported by experimental evidence demonstrating a delayed accumulation and longer half-life of Rex compared to Tax [16]. More recently, we also analyzed the expression kinetics of HTLV-2 mRNAs in chronically infected cell lines and in PBMCs obtained from HTLV-2-infected patients. Results indicated a “two-phase” expression kinetics that is reminiscent of that of HTLV-1. Furthermore, this study revealed that HTLV-2 expresses higher levels of mRNAs encoding inhibitors of Tax and Rex, that is, p28 and truncated isoforms of Rex (tRex), respectively (Bender et al., submitted), suggesting that HTLV-2 may be characterized by a more latent expression profile, compared to HTLV-1.

## 3. Functional Comparison of Tax-1 and Tax-2

HTLV-1 and HTLV-2 Tax (Tax-1 and Tax-2) are required for viral replication, acting as transactivators of proviral transcription from the 5′LTR. Tax may also enhance HBZ transcription through the 3′LTR, although this effect is weaker compared to that on the 5′LTR [18, 19].

**Figure 2:**
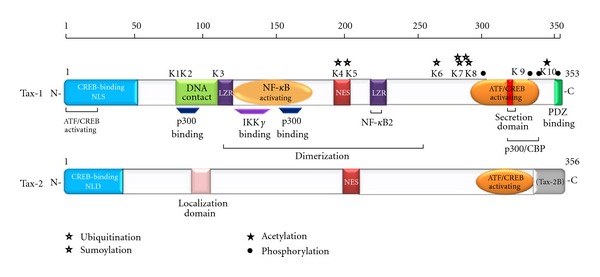
Schematic representation of Tax-1 and Tax-2 structural and functional domains. The positions of the amino acids modified by ubiquitination, sumoylation, acetylation, and phosphorylation are indicated.

In addition to these effects on viral transcription, Tax has a pivotal role in the immortalization and transformation of infected cells by enhancing the expression of cellular genes that control T-cell proliferation and by interacting with proteins that control mitotic checkpoints and inactivate tumor suppressors pathways [20–23]. Tax-1 and Tax-2 present an overall structural and functional homology, although some domains and activities appear to be distinct in the two proteins [24, 25]. Tax-1 is a 353 amino acids (aa), 40 kDa protein, highly conserved in all HTLV-1 serotypes. Tax-2 has been characterized mainly from HTLV-2 subtypes A and B [26]. Tax-2B has 356 aa residues, whereas Tax-2A presents a 25 aa C-terminal truncation. Tax-1 and Tax-2B share an amino acid similarity of 85% and have several common domains including a cyclic AMP response element-binding protein, (CREB-) binding domain, a zinc-finger, domains required for interaction with proteasomal subunits, with transcriptional coactivators and with proteins involved in transcription, cell cycle progression, and in cell signaling regulation [5]. The C-terminal region of both Tax-1 and Tax-2 includes a CREB-activating domain [5, 27]. The central portion of Tax-1 includes two leucine zipper-like regions (LZR), necessary for DNA interaction and for protein dimerization [28, 29], binding domains for proteins involved in chromatin remodeling, cell cycle control, NF-*κ*B activation, and p300 binding. The main structural difference between Tax-1 and Tax-2 is represented by the lack in Tax-2 of a leucine zipper region which is responsible for noncanonical NF-*κ*B activation [30–32] and of the C-terminal motif, which mediates association with protein containing PDZ domains [24] (Figure 2).

 Based on the initial studies on the subcellular localization of Tax-1 and Tax-2 indicating that Tax-1 was localized in the nucleus [33] and Tax-2 in the cytoplasm [34], detailed studies have been devoted to the characterization of the nuclear localization signal of both proteins. A nuclear localization signal (NLS) has been mapped to the first 60 aa of Tax-1 [35, 36] and in the first 42 aa of Tax-2 [37]; an additional NLS, located at position 89–113 of Tax-2, is responsible for the divergent cellular localization compared to Tax-1 [34].

Tax-1 localizes mainly in nuclear bodies in which two subunits of NF-*κ*B (p50 and RelA) are present [38]; in the cytoplasm, Tax-1 localizes in organelles associated with secretory pathways [39], in structures associated to the microtubule organizing center (MTOC) [40], and in the cell to cell contact regions termed virological synapses [41]. Interestingly, the posttranslational modification of Tax is tied to its subcellular localization and ability to activate the NF-*κ*B pathway, a key step in HTLV-1-mediated transformation. In particular, ubiquitinated Tax binds and colocalizes IKK subunits at a centrosome-associated signalosome leading to the release of active IKK [42, 43]. Using live-cell imaging, Kfoury et al. also showed that Tax shuttles among nuclear bodies and the centrosome, depending on its ubiquitination and SUMOylation [44]. In contrast, Tax-2 is more abundant in the cytoplasm [34, 37]. Additional functional properties of Tax proteins derive from their effects in the reorganization of TAB2-containing cytoplasmic structures which include RelA and calreticulin [45].

Both Tax-1 and Tax-2 are necessary and sufficient for HTLV-mediated immortalization of primary human T cells [46, 47]. Moreover, Tax-1 induces an ATLL-like T-cell malignancy in transgenic mice [48]. Tax-1 induces IL-2 expression through the transcription factor NFAT in Jurkat cells following stimulation by TPA [49] and upregulates the expression of additional genes encoding cytokines, chemokines, cell surface ligands, and their receptors [24]. Unlike Tax-1, Tax-2 activates IL-2 gene expression through NFAT, even without additional stimulation [24, 50].

Tax-1 alters several cellular signaling pathways by interacting with the cellular transcription factors NF-*κ*B, CREB, serum responsive factor (SRF), and activator protein 1 (AP-1), that control cell proliferation, intracellular protein distribution, cell migration, apoptosis, and genetic stability [51]. More than 100 proteins have been shown to interact with Tax-1 [22]. Comparative studies showed that both Tax-1 and Tax-2 constitutively activate the canonical NF-*κ*B pathway by interacting with RelA and the I*κ*B kinase complex (e.g., IKK*α*, IKK*β*, and NEMO/IKK*γ*) [22, 52]. Interestingly, Tax-1, but non Tax-2, also activates the noncanonical NF-*κ*B by enhancing p100 processing and nuclear translocation of p52 [31]. Tax-1 and Tax-2 also differ in their mechanism of LTR transactivation, as Tax-1 recruits the CBP, p300, and PCAF coactivators factors, whereas Tax-2 does not require PCAF [53].

Compared to Tax-2, Tax-1 shows an overall higher activity as a transactivator and in transformation capacity as well as inhibition of p53 functions [33, 54, 55]. In contrast to Tax-1, Tax-2 is unable to induce micronuclei [56], does not perturb development and maturation of pluripotent hematopoietic progenitor cells [57], and does not induce G0/G1 cell cycle arrest [58].

## 4. Rex-1 and Rex-2

**Figure 3:**
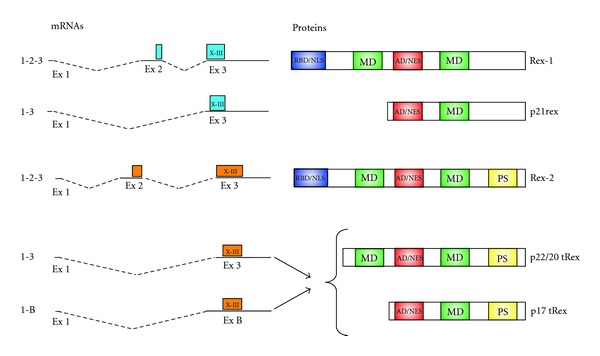
Comparison of the protein isoforms coded by the x-III ORF of HTLV-1 and HTLV-2. Indicated is the exon composition of the mRNAs (left) and the domain structure of the corresponding proteins (right) coded by the x-III ORFs of HTLV-1 and HTLV-2. HTLV-1 produces a single truncated x-III ORF: p21rex; HTLV-2 produces a family of truncated x-III isoforms ranging from 22 to 17 kDa. RBD/NLS: rna binding domain/nuclear localization signal; MD: multimerization domain; AD/NES: activation domain/nuclear export signal; PS: phosphorylation site.

Expression of complex retroviruses is controlled at the posttranscriptional level by viral-encoded regulatory proteins that actively transport intron-containing mRNAs in the cytoplasm. In HTLVs, this function is carried out by Rex, that is produced by a doubly spliced dicistronic mRNA that also encodes Tax. HTLV-1 Rex (Rex-1) is a 27 kDa, 189 aa protein; HTLV-2 Rex (Rex-2) is a 170 aa, 26/24 kDa protein that shares 60% homology with Rex-1 at the amino acid level (Figure 3). Both Rex-1 and Rex-2 are phosphoproteins that localize to nucleus, nucleoli, and nucleolar speckles [3, 59–62]. Although mainly detected in the nuclear compartment, Rex actively shuttles between the nucleus and the cytoplasm [60, 63], a property that is intimately linked to its ability to transport incompletely spliced viral RNA form the nucleus to the cytoplasm.

**Figure 4:**
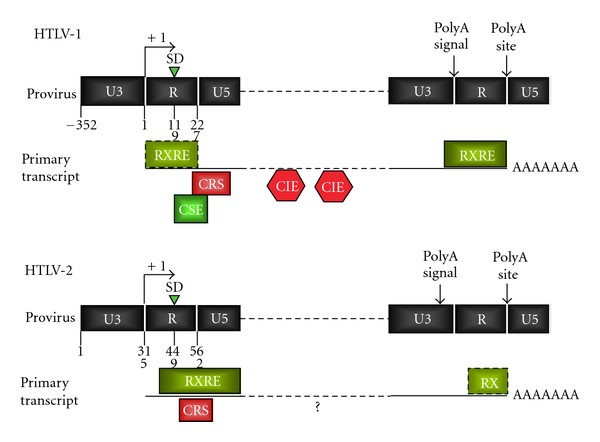
Comparison of the cis acting regulatory elements of HTLV-1 and HTLV-2. Position of the cis acting posttranscriptional regulatory elements of HTLV-1 and HTLV-2 are indicated. RXRE: Rex-responsive element; CRS: cis-acting repressive sequences; CSE: cis-acting stimulatory element; CIE: cis acting inhibitory elements.

 The function of Rex-1 is mediated through binding to a 254-nucleotide stem-loop cis acting RNA element termed the Rex-responsive element (RXRE-1) [64] present in the U3/R region of the LTR. Due to the positions of the transcription start site, major splice donor, and polyadenylation signal/site, the full-length RXRE is located at the 3′ end of all HTLV-1 transcripts, while the 226 nt- Rex-2 responsive elements (RXRE-2), which maps to the R/U5 region, is located at the 5′ end of the unspliced HTLV-2 mRNA (Figure 4) [65–67]. Furthermore, the ability of the RXRE-1 to fold into stem loop structure brings the polyadenylation signal into close proximity to the GU rich polyadenylation site [68, 69] ensuring efficient polyadenylation of viral mRNAs.

 Although Rex is not required for cellular immortalization *in vitro*, it is necessary for infectivity and viral persistence *in vivo* [70], since expression of the viral RNAs encoding the structural proteins is Rex dependent. Therefore, the Rex-RXRE interaction was proposed to act as a molecular switch controlling the transition between productive and latent phases of HTLV-1 infection, an hypothesis that is consistent with results of studies of the kinetics of expression of HTLV-1 mRNA (see above) [16].

 The domain structure of Rex-1 and Rex-2 is similar, with NLS, RNA binding domains (RBD), multimerization domains, and nuclear export signals (NES) (Figure 3). The N-terminal arginine-rich region (amino acids 1–19) acts as nuclear localization signal (NLS) [61, 71] and as RNA binding domain (RBD) which mediates Rex binding to the RXRE [72]. A leucine-rich sequence located near the middle of the protein (Rex-1 aa 79–99; Rex-2 aa 81–94) functions as activation domain (AD) [73] and contains the nuclear export signal (NES) [63, 74]. The NES interacts with the protein chromosome region maintenance interacting protein 1 (CRM1/exportin 1) and allows export of the Rex-viral mRNA complex from the nucleus to the cytoplasm [75]. CRM1 belongs to the importin-*β* family, whose members act as RNA transporters between the nuclear and cytoplasmic compartments [76]. Mutations of the four leucine residues within the NES demonstrate that they are critical for nuclear export of mRNA [63, 74]. In addition, a unique C-terminal domain has been described for Rex-2 that is a target for serine phosphorylation and may also contribute to efficient nucleocytoplasmic shuttling [60]. The two regions flanking the NES (aa 57–66 and 106–124 for Rex-1; aa 57–71 and 124–132 for Rex-2) are required for the assembly of Rex into multimeric structures upon binding to the RXRE [75, 77], a process that is critical for the nuclear export of viral mRNA since multimerization-defective mutants of Rex act as dominant-negative mutants [77]. The multimerization of Rex on the viral mRNAs is also enhanced by CRM1 [78, 79] and by the translation-initiation factor eIF-5A [80].

 Following Rex-RXRE binding and Rex multimerization, CRM1 is recruited into the complex [75], binds to RanGTP, and translocates the Rex-mRNA complex across the nuclear pore by interacting with nucleoporins. In the cytoplasm, RanGTP is then converted to RanGDP and is released from the Rex-mRNA complex. Other proteins affecting Rex function include Ran binding protein 3 (RanBP3), a scaffold protein that stabilizes the RanGTP-CRM1-Rex-mRNA complex in the nucleus [78, 81] and SRC-associated in mitosis 68 (Sam 68), which increases the Rex-mRNA binding in a CRM1-independent fashion [82].

 In addition, to these effects on viral gene expression, Rex-1 enhances Tax-mediated upregulation of IL-2 and stabilizes the IL-2R*α* mRNA [83, 84], thus possibly contributing to transformation of HTLV-1 infected cells. Rex may also enhance the expression of FynB, a src family tyrosine kinase that regulates T-cell receptor stimulation [85, 86] as well as VCAM-1 and LFA-3 [87], two surface molecules important in T-cell adhesion and proliferation. More recently Rex-1 was shown to inhibit the RNA silencing pathway by interacting with Dicer, a member of the RNase III family that converts the double-stranded short hairpin RNA to the single-stranded siRNA (small interfering RNA) [88].

 Furthermore, hnRNP A1 (heterogeneous nuclear ribonucleoprotein A1) impairs the posttranscriptional regulation of HTLV-1 gene expression, by interfering with the binding of Rex to the RXRE [89]. Knockdown of hnRNP A1 expression in the chronically infected T-cell lines C91PL using siRNA increased the levels of the unspliced gag/pol mRNA in the nucleus (2-fold) and cytoplasm (3-fold), while a more modest increase of the tax/rex mRNAs was observed in both compartments and the expression and distribution of the env mRNA was not significantly altered [90].

## 5. Cis Acting Regulatory Elements Important for Rex Function

The expression of the alternatively spliced mRNAs of HTLV-1 and HTLV-2 is controlled by the relative influence of positive and negative sequences present on the primary transcript. Two major types of RNA cis acting elements have been described: (i) the RXRE (described above) which mediates Rex-dependent nuclear export and (ii) cis acting repressive sequences (CRS) that determine poor stability and/or inefficient nucleocytoplasmic export. Although the general layout and function of these elements are similar between the two viruses, several interesting differences can be pointed out (Figure 4) [91–93].

 Studies based on heterologous reporter plasmids mapped a CRS in the U5 region of HTLV-1 [93, 94]. Due to the positions of the transcription start site, major splice donor and polyadenylation signal/site, the HTLV-1 CRS is present only at the 5′ end of the unspliced mRNA (Figure 4). Interestingly, an additional CRS at the 3′ end of all transcripts overlaps the RXRE and acts synergistically with the 5′-CRS. Confocal microscopy analysis indicated that both the 5′ and 3′ CRSs act at the posttranscriptional level, most likely as nuclear retention sequences. Deletion of both the 5′ and the 3′ CRS resulted in constitutive Rex-independent nuclear export and expression of mRNAs. A 5′ CRS acting as a nuclear retention sequence was also mapped in the R-U5 region of HTLV-2 [65]. As the 5′ CRS does not bind Rex-1 [66, 95–97], its inhibitory function is likely to be mediated by other viral and/or cellular RNA-binding proteins.

 A further layer of complexity is added by a study that analyzed the postsplicing steps of HTLV-1 regulation by testing the expression of individual full length viral mRNAs in their intronless form. Results of this study showed that mRNA 1–3 (encoding p21rex), mRNA 1-2-3 (encoding Tax and Rex), and mRNA 1-2-B (encoding p30/tof) were inefficiently expressed when transcribed in their intronless form. The defective expression of these mRNAs was due to the inhibitory activity of the RXRE and the lack of a 5′ intronic region that counteracted the inhibitory effect of the RXRE. This cis acting stimulatory element (CSE) was mapped to the major splice donor and sequences overlapping with, but functionally distinct from, a previously described transcriptional enhancer [98].

 In addition to the CRS, other cis acting inhibitory elements (CIEs) were mapped within the gag-pol and env regions of the HTLV-1 genome [99]. The inhibitory effect of these regions is counteracted by binding of Rex to the RXRE, although it is not clear whether their mechanism of function is mainly at the level of RNA stability or nucleocytoplasmic export. At present, no intragenic CIEs have been described in HTLV-2.

## 6. Truncated Isoforms of Rex

Both HTLV-1 and HTLV-2 produce truncated isoforms of Rex which lack the N-terminal arginine-rich RBD/NLS and are therefore predicted to be incapable of binding the RXRE and accumulate in the nuclear compartment. HTLV-1 produces a single truncated x-III ORF product termed p21rex, which also lacks the oligomerization domains of full-length Rex (Figure 3) [100, 101]. It was hypothesized that p21rex might act as a repressor of full-length Rex, thus inhibiting the expression of virion-associated proteins and playing a role as a latency-inducing factor in the HTLV-1 life cycle [100].

 HTLV-2 produces a family of truncated isoforms ranging from 22 to 17 kDa which differ in the initiation codon usage and phosphorylation status [59]. These truncated x-III ORF products of HTLV-2, that we propose to term tRex, are expressed from two singly spliced mRNAs containing exon 1 linked to either exon 3 or to exon B (Figure 3). Initiation of translation from the first AUG codon located within the x-III ORF gives rise to two major protein isoforms of 22 and 20 kDa and a minor 18-kDa protein differing by posttranslational modification. Translation from the second AUG of the x-III ORF produces a 17-kDa protein [3, 59]. The multimerization and activation domain of Rex2 are also present in the 22–20 and 18 kDa tRex proteins. Cotransfection assays demonstrated that the tRex proteins were able to inhibit the ability of Rex to activate the expression of a Rex-dependent mRNA. Subcellular fractionation studies showed that Rex was preferentially localized in the cytoplasmic or nuclear fraction depending on its phosphorylation status and that coexpression of Rex with tRex changed the phosphorylation pattern of Rex and intracellular localization of tRex [59].

## 7. Expression and Function of Accessory Genes Coded in the X Region of HTLV-1 and HTLV-2

p30tof and p28 are products of the x-II ORF of HTLV-1 and HTLV-2, respectively. p30tof is coded by a doubly spliced mRNA containing exons 1, 2, and B; p28 is coded by 2 singly spliced mRNAs containing exons 1 and 3 or exons 1 and B (Figure 1). The two proteins are both dispensable for viral propagation *in vitro* but important for viral spread and persistence in animal models [102–104]. p30tof and p28 share some functional analogies as they both sequester the tax/rex mRNA in the nucleus, thus reducing viral expression, an effect that may result in viral latency and blunt immune recognition of infected cells [105, 106]. By interacting with the coactivator CBP/p300 [107, 108], p30tof also affects Tax-mediated transcription of viral and cellular genes at the transcriptional and posttranscriptional levels, including genes involved in T-cell activation and apoptosis [109, 110]. Further studies revealed that p28 function was partly distinct from that of p30tof in that it was devoid of transcriptional modulating activity. Moreover, p30tof interacts with the RNA-binding domain of Rex inhibiting Rex binding to the RxRE [111, 112]. By interacting with PU.1, p30tof inhibits its transcriptional activity, resulting in the downregulation of Toll-like receptor 4 (TLR4) expression from the cell surface [113], suggesting that the protein might also play a role in reducing activation of adaptive immunity in HTLV-1-infected patients.

 p13, a short isoform coded by the x-II ORF of HTLV-1 from a singly spliced mRNA containing exons 1 and C (Figure 1), corresponds to the C-terminal 87 amino acids of p30tof [11] and is localized mainly in the mitochondrial inner membrane [114] and in part to the nucleus [115, 116]. p13 increases mitochondrial permeability to K^+^ and activates the electron transport chain, resulting in increased mitochondrial reactive oxygen species (ROS) production [117]. These changes in ROS homeostasis affect both cell survival and proliferation depending on the cell's inherent ROS set-point. While in normal resting T-cells, which have low ROS levels, p13 expression resulted in mitogenic activation, in cancer cells which are characterized by a high ROS setpoint, p13 induced cell death [114, 115, 118–121]. So far, no HTLV-2 ortholog of HTLV-1 p13 has been identified.

 HTLV-1 p12 and p8 are coded by the x-I ORF from a singly spliced mRNA containing exons 1 and A or exons 1 and B (Figure 1). p12 localizes in the endoplasmic reticulum (ER) and in the Golgi apparatus, where it interacts with the *β* and *γ*_c_ chains of the interleukin-2 receptor (IL-2R), reducing their surface expression [122]. p12 expression results in activation of STAT-5, which provides a mitogenic signal to T-cells [123]. p12 also decreases surface expression of MHC-I, thus contributing to blunt lysis of HTLV-1-infected cells by CTL [124]. p12 also interacts with calreticulin and calnexin [125] resulting in increased Ca^2+^ release from the ER [126] and activation of the NFAT, a mitogenic pathway in T cells [127–129]. Within the ER, p12 is cleaved into an 8 kDa protein (p8) which traffics to the immunological synapse and favours T-cell anergy. p8 also increases cell-to-cell viral transmission through the formation of intercellular conduits among T cells [130, 131]. In analogy to HTLV-1 p12, HTLV-2 p10 and p11 were shown to bind the MHC heavy chain; however, p10 and p11 did not bind other targets of p12, such as the IL2R *β* chain or the 16-kDa subunit of the vacuolar H+ ATPase [132]. No functional homologue of p8 has been described in HTLV-2.

## 8. Functional Comparison of Minus-Strand Genes

Transcription from a promoter in the 3′ LTR generates transcripts from the complementary strand which encode the HBZ (HTLV-1) and APH-2 (HTLV-2) genes [12, 13]. The negative strand of the HTLV-1 genome [133] generates at least 2 different transcripts, one spliced (hbz sp1) and the other unspliced (hbz us) [134–136]. Studies in infected cells indicated that hbz sp1 was more abundant than hbz us [137]. Hbz sp1 has multiple transcriptional initiation sites in the U5 and R regions of the 3′ LTR, whereas transcription of the hbz us mRNA initiates within the tax gene. Both hbz sp1 and hbz us promoters are TATA-less [19]. The transcription of hbz sp1 is dependent on the Sp1 transcription factor and is weakly responsive to Tax [19, 138], a finding that is consistent with the fact that hbz is expressed in HTLV-1-infected cells regardless of the expression levels of Tax and hbz expression exhibits a stronger correlation with provirus load than tax expression. Hbz sp1 is translated into a protein of 206 aa, while hbz us produces a protein of 209 aa differing by 7 aa at the N-terminus [135]. The domain structure of the HBZ protein includes an N-terminal transcriptional activation domain (AD), a central domain (CD), and a C-terminal basic ZIP domain (bZIP) [12]. The HBZ SP1 protein is more abundant and has a longer half-life than the US isoform [19]. HBZ US protein mainly localizes in nuclear bodies while HBZ-SP1 is mainly nucleolar; two regions rich in basic amino acids and a DNA binding domain are associated with nuclear localization of HBZ [139, 140].

HBZ is not necessary for *in vitro* transformation but increases infectivity and viral persistence in *in vivo* animal model [141]; furthermore, HBZ was demonstrated to be oncogenic in a transgenic mouse model [142]. HBZ interacts with a number of transcription factors. Through interactions mediated by its bZIP domain, HBZ inhibits the ability of CREB-2 to bind to the HTLV-1 LTR, resulting in the inhibition of Tax-mediated transcription from the 5′ LTR [12]. HBZ interacts with the KIX domain of CBP/p300 via LXXLL-like motifs in its N-terminal region, leading to suppression of viral transcription by inhibiting the recruitment of CBP/p300 to the 5′ LTR promoter [143]. In addition, by forming a trimeric structure with Smad3 and p300, HBZ enhances TGF-*β* signalling [144] resulting in upregulation of Foxp3, a marker characteristic of HTLV-1 infected cells as well as of Treg cells [142].

 HBZ's bZIP domain also mediates formation of heterodimers with c-Jun, JunB, and JunD [145, 146]. Binding of HBZ to JunB and c-Jun decreases their DNA binding activity by preventing their interaction with Fos, leading to repression of the AP-1 complex [147]. Furthermore, HBZ mediates proteasomal degradation of c-Jun [147] and sequestration of JunB in nuclear bodies [140]. In contrast, the interaction of HBZ with Jun-D results in the activation of JunD-dependent cellular genes, including hTERT, the catalytic subunit of human telomerase [146, 148]. Through its N-terminal region, HBZ interacts with IRF-1 (interferon regulatory factor 1) reducing its DNA-binding ability and its stability, resulting in a reduction of IRF-1-mediated apoptosis [149]. HBZ inhibits the classical NF-*κ*B pathway by two different mechanisms: by inhibiting the DNA binding of the NF-*κ*B subunit p65 and by increasing the expression of PDLIM2, the E3 ubiquitin ligase of p65, leading to enhanced ubiquitination and degradation of p65 [150]. The downregulation of the NF-*κ*B pathway was proposed to counteract the onset of Tax-induced cellular senescence, which results from hyperactivation of the NF-*κ*B pathway [151]. HBZ expression is associated with proliferation of ATLL cells *in vivo* and *in vitro* [136, 152]. Mutational analyses showed that the hbz mRNA, rather than HBZ protein, has a growth-promoting effect on T cells [136] possibly by up-regulating the transcription of the E2F1 gene and its downstream targets. Interestingly, only hbz sp1, not hbz us, promotes proliferation of T-cells, indicating that the first exon of the hbz sp1 transcript is critical for this activity [19]. Furthermore it has been shown that hbz sp1 mRNA promotes Tax expression by repressing the p30tof, an inhibitor of the tax/rex mRNA; interestingly, this effect is not mediated by the 5′ stem-loop structure of hbz [153]. Moreover, the recent finding that over 90% of the hbz transcripts are localized in the nucleus in the chronically infected cell line C91PL and in HeLa cells transfected with the ACH HTLV-1 molecular clone underscores their importance as noncoding RNAs [16].

 Following the discovery of HBZ, an antisense transcript was identified in HTLV-2 [13]. Its product, coded by an ORF located between Tax and Env, was named APH-2. Although the APH-2 mRNA was detected in HTLV-2 infected cell lines and in HTLV-2-infected patients, a quantitative comparison of the APH-2 expression levels with proviral loads has not been reported so far. The APH-2 mRNA is transcribed from the R and U5 regions of the 3′-LTR, spliced, and polyadenylated. APH-2 is a 183-aa protein, localized mainly in the nucleus. Despite the lack of a canonical bZIP domain, APH-2 interacts with CREB (but not with CBP/p300) and represses Tax-2-mediated transcription. In analogy to HTLV-1 hbz, also the APH-2 transcript exhibits a marked nuclear localization in chronically infected cell lines (Bender et al., submitted).

## 9. Conclusions and Perspectives

HTLV-1 and HTLV-2 are complex retroviruses with similar genetic structures but significantly different pathogenic potential. Many studies were aimed at defining the viral genetic determinants of these viruses by comparing the expression strategies and functional properties of the different gene products of the two viruses. Although these studies added considerable knowledge to the understanding of the HTLV-1 and HTLV-2 gene expression, important questions still remain open. The Tax proteins of the two viruses, although showing many similarities, exhibit some differences, especially regarding their capability to activate the NF-kB pathway and to induce genetic instability. The mechanism of function of HBZ and APH-2 as noncoding RNAs, which was underscored by their predominant nuclear localization [16], also deserves further studies; in particular, it would be interesting to test whether, in analogy tho HBZ, APH-2 may promote T-cell proliferation and exhibit pathogenic properties in transgenic mice. Although most of the accessory proteins appear to be orthologues in the two viruses, p13 and p8 are peculiar of HTLV-1, while p11 of HTLV-2 does not appear to have an HTLV-1 orthologue. Further studies should be aimed at testing whether the functions of these proteins, along with the differences in Tax-1 and Tax-2, might contribute to explain the different pathogenic potential of HTLV-1 and HTLV-2. In both viruses, the Rex-dependence of viral mRNA is determined by cis acting inhibitory sequences that decrease mRNA stability and nuclear export. A thorough analysis of the mechanism of function of these elements, for example, through a definition of their protein binding partners, is likely to shed light into the mechanisms controlling mRNA turnover and nucleocytoplasmic trafficking. The complex and partly redundant splicing pattern of deltaretroviruses also raises the question as to which mechanism(s) determine splice site selection, resulting in distinct patterns of viral gene expression and, possibly, different clinical outcomes of HTLV infection.

## Figures and Tables

**Table 1:** Regulatory and accessory proteins coded by HTLV-1 and HTLV-2. The proteins with recognized functional analogies in the two viruses are indicated in bold. p8 and p13 proteins (indicated in bold italic) are unique to HTLV-1; p11 (indicated in italic) is unique to HTLV-2.

	ORF x-I	ORF x-II	ORF x-III	ORF x-IV	ORF x-V	Minus strand
HTLV-1	p12, ***p8***	**p30, *p13 ***	**Rex, p21Rex**	**Tax**	**—**	**HBZ**
HTLV-2	p10	**p28**	**Rex, tRex**	**Tax**	*p11*	**APH-2**
